# Effectiveness of Fixed Dose Radioactive Iodine (RAI) for the Treatment of Hyperthyroidism: Experience of a Teaching Hospital in South West Nigeria

**DOI:** 10.4274/Mirt.08370

**Published:** 2013-08-01

**Authors:** John Enyi Ejeh M, Karounwi Omotayo Ogunjobi, John Enyi Ejeh, Kayode Solomon Adedapo, Joshua F Eniojukan

**Affiliations:** 1 University of Ibadan, University College Hospital, Department of Nuclear Medicine, Ibadan Nigeria; 2 University of Ibadan , College of Medicine, Department of Chemical Pathology, Ibadan Nigeria; 3 Niger Delta University, Faculty of Pharmacy, Yenagoa, Nigeria

**Keywords:** I-131, radioisotope therapy, Hyperthyroidism, treatment effectiveness, Nigeria

## Abstract

**Objective:** Using radioactive iodine (RAI) as the first line therapy for Graves’ hyperthyroidism and as the treatment of choice for relapsed Graves’ disease is increasing in recent times. However, there has been little consensus on the most appropriate dose to use. So this study is to determine the response of hyperthyroidism to fixed doses of 370 MBq and 555 MBq RAI therapies and determine the incidence of hypothyroidism at 6 months post therapy.

**Methods:** Hyperthyroid patients’ case records treated with radioiodine was retrospectively reviewed to determine the response rate of hyperthyroidism to the two fixed dose regimens. Statistical analysis was done with SPSS version 15.0 and the level of statistical significance was taken as p<0.05. Forty subjects, 6 males (15%) and 34 females (85%) received RAI therapy for Graves’ hyperthyroidism, mean age was 49.4 years (range, 25-75years). The thyroid function status at 6 months post therapy was available for all subjects. 24 patients (60%) received 370 MBq while 16 patients (40%) received 555 MBq.

**Results:** The response for fixed doses of 370 MBq and 555 MBq were similar (100%). Also, the incidence of hypothyroidism in these subjects which was 66.6% with fixed dose of 370 MBq and 62.5% with fixed dose of 555 MBq within 6 months post RAI therapy were similar.

**Conclusion:** SRAI is highly effective for the treatment of hyperthyroidism, with a cure rate of 100%. However, it has proved impossible to determine a fixed dose regimen for individual patients accurately to guarantee an euthyroid state. This is because hypothyroidism is a natural predictable sequel of RAI therapy.

**Conflict of interest:**None declared.

## INTRODUCTION

Radioactive iodine (RAI) therapy is employed in the treatment of various thyroid disorders. In Graves’ disease, RAI has achieved superior cure rates compared with anti-thyroid drugs (thionamides) and surgery when measured by proportions of patients who remained euthyroid or hypothyroid for at least 1 year. Therefore, there has been an increase in the use of radioiodine as first line therapy for Graves’ hyperthyroidism and the treatment of choice for relapsed Graves’ disease (1,2). While the various treatment modalities for patients with Graves’disease would continue to be diverse, (3,4) our emphasis, however, is on radioiodine therapy. The logical treatment for Graves’ disease would be to remove the cause, which is the thyroid- stimulating immunoglobulin (TSI) (5,6).

Several reviews have covered the use of anti-thyroid medications (7,8,9,10) whose treatment goal is to provide symptomatic relief until definitive treatment can be effected. Surgery as an option predates the use of the less invasive radioisotope therapy, but is still required in cases where the thyroid gland is enlarged and is causing compression to the neck structures, or when the underlying cause of the goiter may be cancerous in origin. Extensive experience has established RAI as an effected, practical, and inexpensive agent to permanently control hyperthyroidism. 

The aim of radioiodine treatment is to destroy sufficient thyroid tissue and to cure hyperthyroidism by rendering the patient either euthyroid or hypothyroid. Although it is highly effective, with a cure rate approaching 100% after one or more treatments (11), it has proved impossible to titrate doses for individual patients accurately to guarantee euthyroid status (12). Despite more than half a century of experience, there is little agreement regarding the most appropriate dose regimen (2,13). Regimens used have included low doses below 185MBq (5mCi) (11,14,15) various fixed doses 185MBq (5mCi) to 555MBq (15mCi) (11,15,16,17,18) and doses based on dosimetric calculations determined on gland size (19). Despite these potential benefits of calculated doses, there is difficulty/failure to demonstrate improvements in cure rate over fixed doses (17,19,20). Furthermore, there is little evidence that using a calculated dose has any advantage over a fixed-dose regimen, in terms of preventing hypothyroidism (21). Many centers prefer the use of a single fixed dose (22).

The influence of anti-thyroid drugs on outcome of radioiodine treatment has also received attention. Some studies have suggested relative radio-resistance in those prescribed anti-thyroid drugs before or after radioiodine (23,24), but others have shown no effect (18) or an effect confined to propylthiouracil (25).

The ideal radioiodine dose regimen remains controversial, and uncertainties persist regarding the influence of underlying disease processes and the effect of treatment with anti-thyroid drugs upon outcome (1). To this end, a retrospective study of all cases of Graves’ hyperthyroidism treated with radioiodine at our center was carried out to evaluate the treatment outcome in these patients and to further optimize radioiodine treatment for individual patients with hyperthyroidism at this center.

## MATERIALS AND METHODS

**Patients**

In this study, 40 patients with Graves’ hyperthyroidism, comprising of 6 males and 34 females were included. Graves’ disease diagnosis was made based on the presence of one of the following: diffuse goiter, ophthalmopathy, and TSH receptor antibodies. The study covered the audit of the case records of patients who had received treatment for Graves’ hyperthyroidism in our center from July 2006 to December 2009. The study evaluated the response or cure rate of hyperthyroidism to fixed doses of 370MBq (10mCi) and 555MBq (15mCi) RAI therapy. It was further done to determine the optimum effective fixed dose 370MBq (10mCi) or 555MBq (15mCi) of RAI therapy for hyperthyroidism and the incidence of hypothyroidism within 1 year of post RAI therapy. Finally, the study determined the effect of pre-treatment with thyrostatic medications on RAI therapy.

**Exclusion Criteria**

Patients who had received radioiodine for hyperthyroidism before July 2006 were excluded from the study. Similarly, patients lost to follow up within 6 months of I-131 therapy or whose records were incomplete were also excluded. We also excluded patients with nodular goiter. The study was therefore, limited to 40 patients with Graves’ hyperthyroidism.

**Method**

Baseline characteristics of patients obtained included: age, gender, presence of eye disease (exophthalmos), the size and type of goiter, duration of hyperthyroidism, pre-treatment with anti-thyroid drugs before RAI therapy, period of time- off therapy before RAI administration. 

The size and type of goiter at diagnosis was categorized on the basis of physical examination by our clinicians as; none (gland impalpable or normal size), small (thyroid palpably enlarged but not visible), and medium or large (palpable and visible goiter) and the volume confirmed by derivation from the ultrasound measurements of length (L), breadth (B) and width (W) all in centimeters. The derived thyroid volumes obtained were the sum of the individual volumes of both thyroid lobes. 

Thyroid imaging or scan with pertechnetate (TcO_4_) was performed on all subjects. Thyroid function tests such as TSH, plasma concentrations of free thyroxine (fT4), and free triiodothyronine (fT3) were performed on the subjects at diagnosis and before taking RAI (131I) by radioimmunoassay (RIA) technique using Wallac WIZARD 1470 Gamma Counter (Perkin Elmer, Turku, Finland).

**Treatment Protocol**

The policy at our center, over the period of the study, was to implement a standard protocol for radioiodine treatment of hyperthyroidism. The use of anti-thyroid drugs and intake of seafood were stopped 5 days before radioiodine therapy and not recommended for a minimum of 1 week after therapy. Patients were assessed and counseled by the nuclear medicine physician 1 week before I-131 therapy. The doses of I-131 prescribed to the patients were empirical (fixed doses of 370-555MBq [10- 15mCi]). However, it is difficult to administer the exact dose (activity) to our patients due to radioactive decay. As a result, twenty-four of our patients received 370MBq (10mCi) RAI (range 9-12mCi) while sixteen received 555MBq (15mCi) (range 13-17mCi). This was because of factors such as; the decay of radioactive iodine, non availability of RAI locally, hence, importation, delay in clearing the consignment from the port as well as delay on the part of patients in coming for RAI therapy. As a result, higher activity of I-131 were ordered to compensate for radioactive decay.

All the patients treated had stopped anti-thyroid medication 5 days before receiving RAI therapy. Thyroid function test results were monitored during subsequent follow up and thyroid status was assessed 6 weeks, 3 months, 6 months after radioiodine administration for all subjects.

**Outcome of Treatment**

Patients were judged to be euthyroid if serum TSH concentrations were within the normal range (0.21-6.0 mIU/L); hyperthyroid if TSH <0.2 mIU/L, and hypothyroid if TSH >6.1 mIU/L. Outcome after radioiodine therapy was defined as the number of doses of radioiodine required to cure hyperthyroidism by rendering patients either euthyroid or hypothyroid. Relapse was defined as clinical and biochemical persistence of hyperthyroidism within 12 months. 

**Statistical Analyses**

All statistical analyses were performed using the statistical package for the social sciences (Windows version 15.0; SPSS Inc, Chicago (IL), US). The chi-square test was used to test for relationship between the two categorical variables.

## RESULTS

Forty patients, 6 (15%) males and 34 (85%) females were enrolled. The female to male ratio was 5.7: 1, showing a higher incidence of Graves’ hyperthyroidism in female than male subjects. The mean age of the group was 49.4 years (range 25- 75 years). There were fourteen younger patients (below 45 years) and twenty-six patients were above 45 years. The incidence of Graves’ hyperthyroidism is higher in older patients than in younger patients. Only four (10%) of the forty patients had had previous thyroidectomy (recurrent toxic goiter) before the radioiodine treatment. Twelve subjects had exophthalmos while twenty-eight were free from ophthalmopathy (Table 1).

Co-morbidity included; thyrotoxic heart failure -2 subjects, hypertension -12 subjects. Thirty-eight patients (95%) had received anti-thyroid drugs; on carbimazole only for more than 2 weeks before treatment. Only two patients (5%) had beta-blocker (propnanolol) alone before receiving RAI (Table 1). The patients were grouped into two fixed doses based on the dose prescribed by the clinician. 

**Figure 1:**
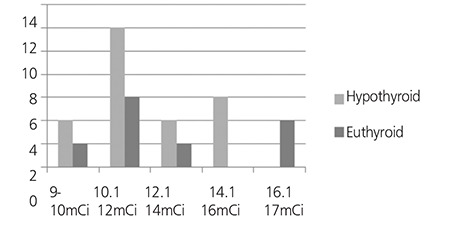
Distribution of patients by radioiodine doses

Twenty-four patients (60%) were treated with an average dose of 370MBq (10mCi), range (9-12mCi) and sixteen patients (40%) received an average dose of 555MBq (15mCi), range (13-17mCi) of radioactive iodine (Figure 1 and Table 2).

The response or cure rate (becoming hypothyroid or euthyroid) at 6 months post RAI was similar (100%) for both doses of 370MBq and 555MBq of radioiodine (Table 3). Also, the incidence of hypothyroidism (TSH > 6.1 mIU/L) was 66.6% for patients who had received 370MBq (10mCi) and 62.5% for those that received 555MBq radioiodine therapy. There was no significant difference in the rate of response or cure of hyperthyroidism for patients who received 370MBq and those that received 555MBq of RAI therapy (Table 3 and Figure 1). 

## DISCUSSION

The use of radioiodine in hyperthyroidism is increasing, particularly as first line therapy for Graves’ disease - where the likelihood of success with anti-thyroid drugs is modest. Furthermore, younger patients are now offered radioiodine earlier in the course of their disease (26), because evidence suggests that onset of Graves’ hyperthyroidism at a young age is associated with increased likelihood of relapse after medical treatment (12,27,28). Over the last 30 years, much attention has focused on achieving euthyroidism by adjusting the dose of radioiodine. Although low fixed doses 185MBq (5mCi) and below are associated with a reduced early incidence of hypothyroidism, they often result in unacceptably low cure rates. Moreover, the development of long-term hypothyroidism seems to be inevitable, irrespective of the amount of radioiodine administered (11,26). Some clinicians now prefer to give a large ablative dose of 555MBq and upwards, which results in early hypothyroidism, so that the need for long-term follow-up of thyroid function in euthyroid patients is obviated.

This study showed that the use of anti-thyroid drugs (carbimazole only) within 2 weeks before or after radioiodine administration was not significantly associated with failure of response to fixed doses of 370MBq and 555MBq. This was because, all the thirty-eight patients (95%) that received pre-treatment with carbimazole responded well to RAI therapy. The contribution of anti-thyroid drugs, therefore, may be clinically relevant at lower doses of RAI therapy (below 185 MBq) and only with propylthiouracil. 

Outcome after radioiodine treatment was defined as the number of doses of radioiodine required to result in cure of hyperthyroidism (euthyroid or hypothyroid). The data from this study demonstrated similar response (100%) to a single fixed dose of radioiodine of 370MBq and 555MBq to achieve cure of hyperthyroidism. In this study the incidence of hypothyroidism is 66.6% with patients who had received 370MBq and 62.5% with those that received 555MBq radioiodine therapy. There was no significant difference or evidence that the incidence of hypothyroidism is lower with fixed dose of 370MBq than 555MBq of RAI therapy and this is in agreement with Allahabadia et al (29). The hypothyroidism observed in the group treated with 14.1-16mCi and the euthyroidism observed in the group treated with 16.1-17mCi could be due to variations in gland size and severity of the disease in the two groups, although, according to Allahabadia et al., the response to treatment in Graves’ hyperthyroidism is unpredictable and factors postulated to predict outcome have not generally proved clinically useful or being widely adopted in clinical practice, therefore, we cannot categorically give reasons for the outcome observed in these groups (12). 

The results, therefore, demonstrated that a single fixed dose of 370MBq or 555MBq of radioiodine is highly effective in curing Graves’ hyperthyroidism. Contrary to expectation, there was no significant increase in the incidence of hypothyroidism at 6 months, using the larger dose. The incidence of hypothyroidism at 6 months was similar for fixed dose of 370MBq and 555MBq RAI therapy, as a result, any advantage in terms of development of hypothyroidism for the low-dose regimen was lost.

Predictably, hypothyroidism accompanies the administration of RAI with annual incidence rates ranging from 62.5% to 66.6 % for 555MBq and 370MBq RAI therapy, respectively. It may however, manifest many years after the administration of even small doses of RAI. A lifelong follow up is thus necessary after the administration of RAI therapy to allow for the early detection of hypothyroidism, as it may be insidious in presentation. This therefore, supports the argument for an early induction of hypothyroidism as against euthyroidism.

## CONCLUSION

In this study, the effective dose of RAI for Graves’ hyperthyroidism was examined and the incidence of early occurrence of hypothyroidism was determined in our cohort of patients who had received two fixed therapy doses of RAI (370 and 555MBq) from July 2006 to December 2009. There was no significant difference in response to RAI treatment between the two fixed doses of 555MBq and 370MBq RAI therapy. Also, the incidence of hypothyroidism following the administration of two fixed doses of RAI was found to be similar at 6 months after the administration of radioiodine. There was no reduction in response rates to radioiodine in all the thirty-eight (38) subjects pre-treated with anti-thyroid drugs (carbimazole).

## Figures and Tables

**Table 1:**
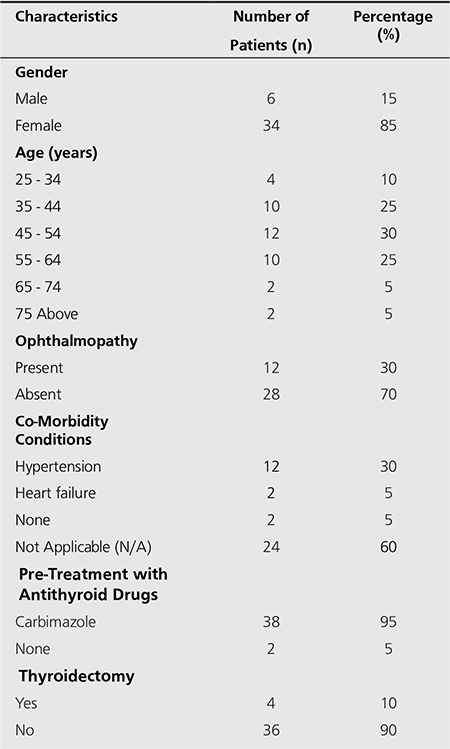
Characteristics of patients with Graves’hyperthyroidism treated with radioiodine

**Table 2:**
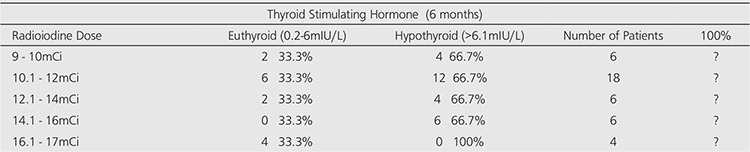
Different Doses of Radioiodine and Response Rate

**Table 3:**
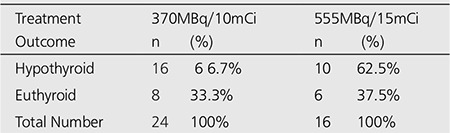
Outcome of a Single Fixed Dose of Radioiodine at 6 months

**Table 4:**
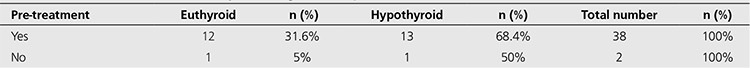
Pre-treatment with Anti-thyroid Drugs and Response after Radioactive Iodine treatment
